# Increasing Participation and Completion Rates in Questionnaire Surveys of Primary Care Patients: Cluster-Randomized Study

**DOI:** 10.2196/67981

**Published:** 2025-02-25

**Authors:** Paul Sebo, Benoit Tudrej, Augustin Bernard, Bruno Delaunay, Alexandra Dupuy, Claire Malavergne, Hubert Maisonneuve

**Affiliations:** 1 University Institute for Primary Care (IuMFE) University of Geneva Geneva Switzerland; 2 University College of General Medicine Université Claude Bernard Lyon 1 Lyon France

**Keywords:** completion rate, missing data, mixed mode, web-based, participation rate, primary care, questionnaire, QR code, tablet, survey, primary care patients, randomized study

## Abstract

**Background:**

Participation and completion rates in questionnaire-based surveys are often low.

**Objective:**

This study aims to assess participation and completion rates for a survey using paper and mixed mode questionnaires with patients recruited by research assistants in primary care waiting rooms.

**Methods:**

This cluster-randomized study, conducted in 2023 in France, involved 974 patients from 39 practices randomized into 4 groups: “paper with incentive” (n=251), “paper without incentive” (n=368), “mixed mode with tablet” (n=187), and “mixed mode with QR code” (n=168). Analyses compared the combined paper group with the 2 mixed mode groups and the “paper with incentive” and “paper without incentive” groups. Logistic regressions were used to analyze participation and completion rates.

**Results:**

Of the 974 patients recruited, 822 (women: 536/821, 65.3%; median age 52, IQR 37-68 years) agreed to participate (participation rate=84.4%), with no significant differences between groups. Overall, 806 patients (98.1%) answered all 48 questions. Completion rates were highest in the combined paper group (99.8%) compared to mixed mode groups (96.8% for paper or tablet, 93.3% for paper or QR code; *P*<.001). There was no significant difference in completion rates between the “paper with incentive” and “paper without incentive” groups (100% vs 99.7%).

**Conclusions:**

Recruiting patients in waiting rooms with research assistants resulted in high participation and completion rates across all groups. Mixed mode options did not enhance participation or completion rates but may offer logistical advantages. Future research should explore incentives and mixed-mode strategies in diverse settings.

## Introduction

Questionnaire-based surveys are valuable tools in a variety of research areas, including health care. For example, they allow data to be collected on patients’ experiences, opinions, and behaviors, which helps health care professionals to better understand their needs and improve the quality of care. Apart from the fact that they are generally inexpensive and take little time, 1 major advantage of questionnaire-based surveys lies in the flexibility they can offer in terms of the mode of administration (ie, face-to-face interviews, telephone interviews, and paper or web-based questionnaires completed at home or elsewhere).

However, to maintain a good level of representativeness and avoid selection bias, it is crucial to obtain high participation and completion rates. For surveys with postal, email, or telephone recruitment, several studies showed that participation rates (especially for web-based questionnaires) [1-4] and completion rates (especially for paper questionnaires) [1,2] tended to be relatively low, whether the participants were individuals, patients, or physicians. Other studies showed that physicians’ participation and completion rates were generally lower than those of the general population, probably mainly because of their workload in the practice and the increasing frequency with which they were asked to respond to surveys [5,6]. Although participation and completion rates are generally particularly low among physicians, these rates may also be suboptimal in studies with patients [7]. This means that the results of a large number of surveys conducted with patients may not be representative of the target population as a whole and may therefore lead to misleading conclusions.

Among the factors likely to influence participation and completion rates, the method chosen to collect data and the use of incentives are key elements [8-12]. Mixed modes, that is, the use of several methods in the same study, seem particularly useful for improving participation rates [9,13]. To our knowledge, QR codes have only been the subject of a few studies, focusing on specific populations and with limited sample sizes [11,14-16]. The usefulness of mixed modes including QR codes to improve participation and completion rates in a questionnaire has not yet been explored in general practice.

The aim of this study was to compare the participation and completion rates for a 48-question survey across 4 groups: a paper questionnaire group with incentive, a paper questionnaire group without incentive, and 2 mixed mode groups (ie, paper or web-based with tablet, and paper or web-based via QR code). Specifically, we analyzed differences between the combined paper group and the 2 mixed mode groups, as well as between the “paper with incentive” and “paper without incentive” groups. Patients were recruited into primary care waiting rooms by research assistants who were available to answer their questions if necessary. We hypothesized that both mixed mode options and incentives would improve participation and completion rates.

## Methods

### Design, Setting, and Study Population

This cluster-randomized study carried out in 2023 in the Rhône-Alpes region (France) was part of an environmental health project aimed at profiling different patterns of meat consumers in primary care, with a view to designing brief interventions to reduce meat consumption.

We used a professional register of primary care physicians from which we randomly extracted 200 physicians using computer-generated random numbers. Five research assistants contacted each randomly selected primary care physician by email until the required number of participating physicians (ie, n=40, Sample Size Determination section) was reached. If the primary care physician refused to participate or did not respond after 3 consecutive reminders, we contacted the next practice on the list.

The research assistants in this study were postgraduate residents in primary care medicine, conducting this work as part of their MD thesis. They had prior clinical experience, which enabled them to effectively interact with patients. To ensure consistency in patient recruitment and minimize variability, all research assistants underwent standardized training before the study began. This training included guidance on presenting the study, explaining the information sheet, and following ethical protocols. Although the study covered a large geographic area, requiring assistants to work autonomously during the recruitment phase, their approach was standardized and aligned with the study’s protocols. Due to logistical constraints, the assistants were not randomly assigned to specific clinics.

We used simple randomization to allocate our sample of medical practices into 4 groups using computer-generated random numbers. Fifteen practices were randomized into the “paper without incentive” group, 10 into the “paper with incentive” group, 7 into the “paper or web-based with tablet” group (ie, patients were given the option of using either paper or a tablet provided by the researchers), and 7 into the “paper or web-based via QR code” group (ie, patients were given the option of using either paper or a QR code if they had their smartphones with them). We used computer-generated random numbers to determine the day of the week on which the study would be carried out in each practice.

### Ethical Considerations

The study was approved by the Research Ethics Committee of the University College of General Practice, Claude Bernard Lyon 1 University (2023-01-03-01). The committee granted approval for the study protocol, including patient recruitment and data collection procedures. No additional ethical approvals were required beyond this institutional review. Participants were nonurgent, French-speaking, consecutive adult patients who were able to understand the study and provide written informed consent. They were informed about the study’s objectives, data confidentiality, and their right to withdraw at any time without consequences. No personally identifiable information was collected, and all data were anonymized before analysis to ensure participant privacy and confidentiality. In line with the environmental health theme of the study, an origami paper containing a seed was provided as an unconditional incentive to all patients in the "paper with incentive" group, regardless of their willingness to complete the questionnaire. No financial compensation was offered to participants. No identifiable features of research participants are present in any images or supplementary materials included in the manuscript.

### Data Collection

Participants were informed by a poster and recruited in the waiting room by a research assistant (20-25 patients per practice) between January 9, 2023, and June 16, 2023. The research assistant verified that the inclusion criteria were met and was available to answer any questions from the participants. The questionnaire (Multimedia Appendix 1) consisted of 48 questions: 4 sociodemographic questions (age, sex, postcode, and occupation), the French version of the feeling of the Sense of Coherence Scale (=17 questions), the French version of the Meat Attachment Questionnaire (=17 questions), and a questionnaire on intentionality adapted from previous studies (=10 questions) [17-19]. The questionnaire generally took about 10 minutes to complete. The paper and web-based questionnaires were designed to be as similar as possible. In the 2 mixed modes (ie, with a tablet or QR code), we included an alert indicating that one or more questions were not answered. However, to be consistent with the paper version, participants did not have to answer all the questions before submitting the web-based questionnaire.

### Statistical Analyses and Sample Size Determination

The sample size calculation was conducted to ensure sufficient power to detect differences in completion rates between groups. For simplification, the calculation was based on comparing proportions between 2 groups. Completion rates reported in the literature vary widely, ranging from 35% to 99%, depending on factors such as the study population, the theme of the study, and the format of the questionnaire [1,2,4]. We hypothesized that the completion rate in our study would be at least 80%, given the presence of a research assistant in the waiting room to assist participants if needed.

We calculated that 428 patients would be required in total to detect a 10-percentage-point difference between groups, with a power of 80% and a significance level of 5%, prior to accounting for clustering [20]. To adjust for clustering within practices, we assumed a conservative intraclass correlation coefficient of 0.02, based on findings from prior research in primary care settings [21], and an average cluster size of 20 patients per practice. This resulted in a design effect of 1.38, inflating the total adjusted sample size to 591 patients. With an average cluster size of 20 patients, this corresponded to 30 clusters in total. As 3 groups were compared, the sample size was increased to 40 clusters to ensure sufficient power.

We computed the proportion of patients who agreed to take part in the study (participation rate), the median number of questions answered with IQR, and the proportion of patients who answered all the questions (completion rate).

To assess whether the participation rate differed between groups, we used logistic regressions adjusted for intracluster correlation within practices. Comparisons were made between the combined paper group, the “paper or tablet” group, and the “paper or QR code” group as well as between the “paper with incentive” and “paper without incentive” groups. Multivariable analyses were not conducted for participation rates, as sociodemographic data were not collected for patients who refused to participate, in accordance with the ethics committee’s recommendations. This limitation prevented us from assessing variations in participation rates by sociodemographic factors.

For the completion rate, exact logistic regressions adjusted for intracluster correlation were used to address sparse data issues, where cells formed by the outcome and categorical predictor variables had few or no observations [22]. Comparisons were made for (1) the three groups based on initial randomization (ie, combined paper, “paper or tablet,” and “paper or QR code”); (2) groups based on the questionnaire format chosen (ie, paper, “web-based with tablet,” and “web-based with QR code”); (3) the “paper with incentive” and “paper without incentive” groups; and (4) sex and age group (<40, 40-60, and ≥60). Multivariable analyses were also carried out, adjusting for sex, age group, and occupation.

All analyses were carried out with STATA (version 15.1; StataCorp LLC).

## Results

**Figure 1:**
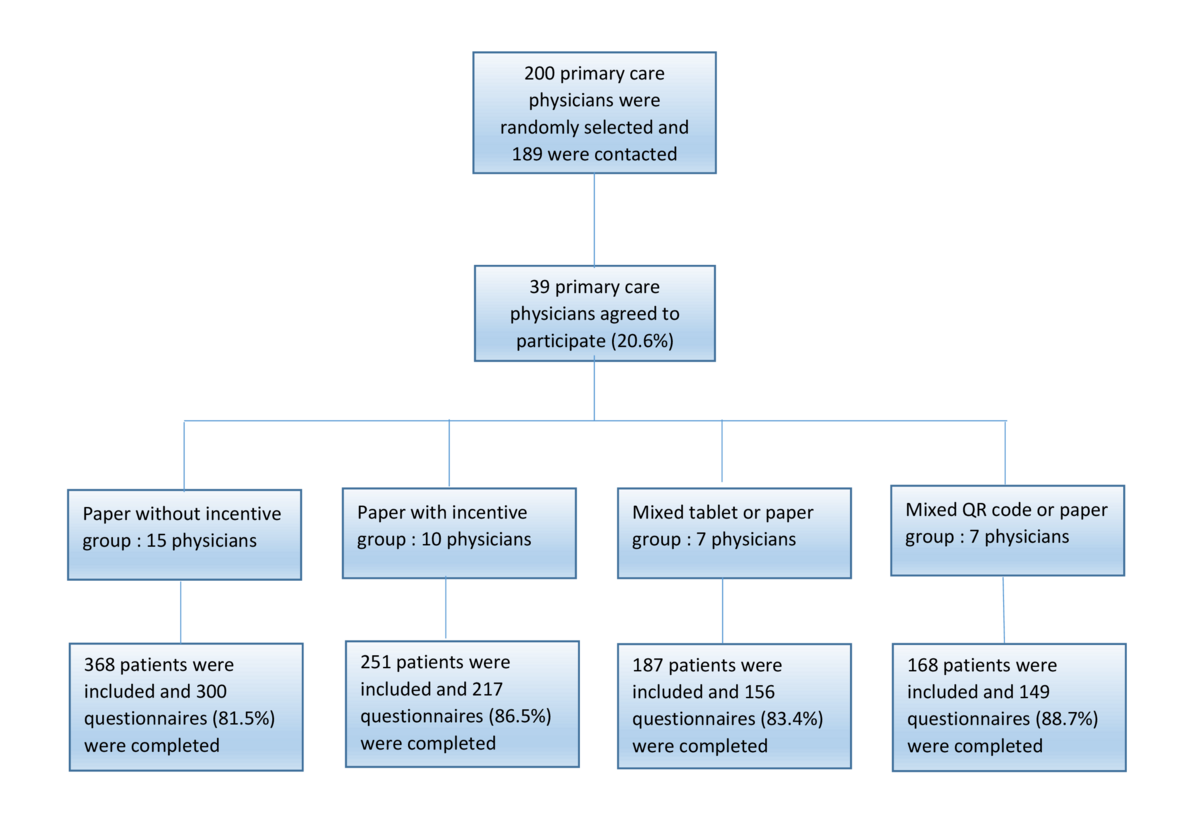
Flowchart of the study.

The flowchart for the study is shown in Figure 1. From the list of 200 primary care physicians, 189 were contacted, of whom 39 (20.6%) agreed to participate. The study included 15 primary care physicians in the “paper without incentive” group, 10 in the “paper with incentive” group, 7 in the “paper or web-based with tablet” group, and 7 in the “paper or web-based via QR code” group.

The study sample consisted of 974 consecutive nonurgent patients who were recruited from these 39 medical practices, representing an average of 25 patients per practice (min=16, max=37). There were 251 patients in the “paper with incentive” group, 368 in the “paper without incentive” group, 187 in the “mixed mode with tablet” group, and 168 in the “mixed mode with QR code” group (Figure 1). Of these patients, 822 agreed to participate in the study, resulting in a participation rate of 84.4% (woman: 536/821, 65.3%; median age of 52, IQR 37-68; min-max=20-93; aged less than 40 years=239/819, 29.2%; 40-60 years=267/819, 32.6%; and more than 60 years=313/819, 38.2%). The distribution of women and men and by age group was similar in all 4 groups (*P* value=0.91 for sex and 0.08 for age group). The majority of participants were retirees (264/822; 32.1%), employees (227/822, 27.6%), and executives or intellectual professionals (166/822, 20.2%). Smaller groups included those not currently active in the workforce (68/822, 8.3%), intermediate professions (41/822, 5%), workers (28/822, 3.4%), artisans or business owners (25/822, 3%), and farmers (3/822; 0.4%).

The main results of the study are presented in Tables 1 and 2. Table 1 shows the differences between the combined paper group, the “mixed mode with tablet” group, and the “mixed mode with QR code” group. The data are organized first by the initial randomization group and then by the chosen questionnaire format. Table 2 focuses on comparing the “paper with incentive” group to the “paper without incentive” group.

**Table 1:** Participation and completion rates and median number of questions answered according to initial randomization group, chosen questionnaire format, sex, and age group^a^.

		Participation rate, n/N (%)	Crude OR^b^ (95% CI)	*P* value^c^		Number of patients having fully completed the questionnaire, n/N (%)		Median number of questions answered (IQR; min-max)	Crude OR (95% CI)	*P* value^d^			Adjusted OR (95% CI)			*P* value^e^		
**Initial randomization group (n=822)**					.23						<.001						<.001	
	Paper (n=517)	517/619 (83.5)	Reference			516/517 (99.8)		48 (48-48; 47-48)	Reference				Reference					
	Paper or tablet (n=156)	156/187 (83.4)	0.99 (0.55-1.80)			151/156 (96.8)		48 (48-48; 10-48)	0.05 (0-0.48)				0.06 (0-0.53)					
	Paper or QR-code (n=149)	149/168 (88.7)	1.55 (0.91-2.63)			139/149 (93.3)		48 (48-48; 21-48)	0.02 (0-0.17)				0.03 (0-0.19)					
**Chosen questionnaire format (n=822)**												<.001						<.001
	Paper (n=634)	N/A^f^	N/A	N/A		633/634 (99.8)		48 (48-48; 47-48)	Reference				Reference					
	Web-based with tablet (n=105)	N/A	N/A	N/A		100/105 (95.2)		48 (48-48; 10-48)	0.03 (0-0.30)				0.03 (0-0.27)					
	Web-based with QR code (n=83)	N/A	N/A	N/A		73/83 (88)		48 (48-48; 21-48)	0.01 (0-0.06)				0.01 (0-0.07)					
**Sex (n=821)**												.20						.17
	Female (n=536)	N/A	N/A	N/A		529/536 (98.7)		48 (48-48; 10-48)	Reference				Reference					
	Male (n=285)	N/A	N/A	N/A		277/285 (97.2)		48 (48-48; 21-48)	0.53 (0.17-1.62)				0.46 (0.14-1.47)					
**Age group (years, n=819)**												.06						.16
	Less than 40 (n=239)	N/A	N/A	N/A		233/239 (97.5)		48 (48-48; 10-48)	Reference				Reference					
	40-60 (n=267)	N/A	N/A	N/A		265/267 (99.3)		48 (48-48; 38-48)	5.06 (1.03-48.62)				3.99 (0.79-39.17)					
	More than 60 (n=313)	N/A	N/A	N/A		308/313 (98.4)		48 (48-48; 21-48	2.35 (0.69-9.08)				1.92 (0.55-7.64)					

^a^The questionnaire consisted of 48 questions: 4 sociodemographic questions, the Sense of Coherence Scale (=17 questions), the Meat Attachment Questionnaire (=17 questions), and an intentionality questionnaire developed by our research team (=10 questions).

^b^OR: odds ratio.

^c^Univariable logistic regression (adjusted for intracluster correlation within practices).

^d^Univariable exact logistic regression (adjusted for intracluster correlation within practices).

^e^Multivariable exact logistic regression (adjusted for sex, age group, occupation, and intracluster correlation within practices).

^f^Participation rates are marked as “N/A” for the chosen questionnaire format, sex, and age group because participants who refused to participate did not indicate their preferred format, sex, or age. Therefore, participation rates, crude odds ratio, and *P* values by these characteristics could not be calculated.

**Table 2:** Comparison of participation and completion rates and median number of questions answered in the “paper with incentives” and “paper without incentives” groups^a^.

	Participation rate, n/N (%)	Crude OR^b^ (95% CI)	*P* value^c^	Number of patients having fully completed the questionnaire, n/N (%)	Median number of questions answered (IQR; min-max)	Crude OR (95% CI)	*P* value^d^
Paper group (n=517)	N/A^e^	N/A	.18	N/A	N/A	N/A	≥.99
Without incentives (n=300)	300/368 (81.5)	Reference		299/300 (99.7)	48 (48-48; 47-48)	Reference	
With incentives (n=217)	217/251 (86.5)	1.45 (0.84–2.49)		217/217 (100)	48 (48-48; 48-48)	1.74 (0-67.75)	

^a^The questionnaire consisted of 48 questions: 4 sociodemographic questions, the Sense of Coherence Scale (=17 questions), the Meat Attachment Questionnaire (=17 questions), and an intentionality questionnaire developed by our research team (=10 questions).

^b^OR: odds ratio.

^c^Univariable logistic regression (adjusted for intracluster correlation within practices).

^d^Univariable exact logistic regression (adjusted for intracluster correlation within practices).

^e^N/A: not applicable.

The participation rate ranged from 81.5% in the “paper without incentive” group to 88.7% in the “mixed mode with QR code” group, but the differences between the groups were not statistically significant. The incentive did not lead to a substantial increase in the participation rate. In this study, 51 patients (32.7%) in the “paper or web-based with tablet” group and 66 patients (44.3%) in the “paper or web-based via QR code” group preferred to complete the questionnaire using the paper version.

Overall, 806 patients (ie, 98.1% of patients) answered all 48 questions on the questionnaire. For the remaining 16 patients, the number of missing data ranged from 1 to 38. These patients were divided as follows: 1 patient belonged to the “paper without incentive” group, 5 to the “tablet” group, and 10 to the “QR code” group. The differences between groups based on the initial randomization and the questionnaire format chosen were small but statistically significant in both univariable and multivariable analyses. By contrast, the differences were not statistically significant for incentive, sex, and age group, but the number of patients with missing data was low.

## Discussion

### Main Findings

This study involved French primary care patients, recruited by research assistants and invited to complete a 48-question survey in the waiting room. We found that the participation rate was over 80% for all groups and that, when given the choice, a number of patients (n=117) preferred to complete the paper rather than the web-based questionnaire. We also found that the completion rate was 99.8% for the paper questionnaire and only slightly less for the web-based questionnaire. Finally, we found that the incentive had no influence on the participation rate or the number of questions answered.

#### Comparison With Existing Literature

Compared with other studies [12,23,24], we achieved excellent participation and completion rates in all groups (for participation rates, much higher than the 60% recommended in some publications [25]), despite a relatively lengthy self-administered questionnaire comprising 48 questions on a sensitive topic. The results of our study tend to reinforce the already known information that, among primary care patients, the response rate may not be affected by the length of the questionnaire, provided that the total duration is less than 15-20 minutes [26,27], which was the case in our study.

We found that the use of mixed options (paper or web-based), although intended to adapt more precisely to the preferences of each participant, did not appear to add value in terms of participation and completion rates. However, the web-based format (or mixed options) may be preferred for other reasons such as improved feasibility. Compared to paper questionnaires, the web-based format generally allows data to be obtained more cheaply (less printing, mailing, or typing costs), more quickly (real-time data tracking and less typing), and more accurately (the structured format minimizes incorrect entries and automatic data transfer minimizes data entry errors) [28]. Alongside the usual methods for collecting web-based data, QR codes have recently come into use in questionnaire surveys. They are simple and effective tools that are known to increase user engagement [29]. In a UK population-based maternity study, the inclusion of QR codes in the survey tended to lead to an increase in response rate, but this effect was limited and was probably also related to other factors (prior notification, short questionnaire, personalized study material, and additional reminder) [8]. In this English study, the initial response rate (30%) was considerably lower than ours.

The results of our study suggest that face-to-face recruitment with a research assistant achieves high participation and completion rates, providing support to participants. Previous studies have already highlighted that this strategy was associated with significantly higher participation rates [7,13,30]. Administering a face-to-face survey in the waiting room has the potential advantage of capturing patients’ experiences when they are most accessible, with fewer competing demands than in other situations.

We found that, in the presence of a research assistant, neither the mixed mode, as discussed earlier, nor the unconditional nonmonetary incentive seemed to have any effect. Nonmonetary incentives are recognized as important factors in improving the recruitment of participants to health-related surveys, with the impact being greater when the incentive is unconditional [31]. In our study, the high participation rate probably prevented us from observing a significant effect in the different methods tested. The presence of a research assistant seems to be the most powerful incentive, probably due to a desirability bias. This could be verified by carrying out the study using the incentives in the absence of a research assistant.

#### Significance and Recommendations for Future Research

This study makes a valuable contribution to the methodology of survey-based research in health care by underscoring the impact of recruitment strategies on both participation and completion rates. The notably high rates we observed across all groups suggest that face-to-face recruitment by a research assistant may be a critical factor for ensuring representativeness, especially in primary care settings where patient engagement can be challenging. Researchers conducting survey studies in similar environments may benefit from prioritizing in-person recruitment with trained personnel, as our findings indicate that this approach effectively mitigates common barriers to participation, such as lack of time. Additionally, while mixed mode survey options (including QR codes) did not significantly increase participation in this study, these tools still offer logistical advantages, such as ease of data collection, reduced error rates, and potential cost savings. For research teams looking to optimize both response rates and operational efficiency, mixed mode strategies may still be worthwhile. Future studies may benefit from investigating the impact of assistant-supported recruitment in different contexts or exploring alternative incentives, such as monetary incentives, that could further enhance response rates, particularly in larger or more geographically diverse samples.

### Limitations

First, the results of our study cannot be generalized in all questionnaire-based surveys. Several studies showed that surveys carried out by recruiting participants by email, post, or telephone often led to mediocre participation rates for the web-based format [1-4] and mediocre completion rates for the paper format [1,2]. In this type of study, mixed mode options and incentives could be useful for positively influencing participation and completion rates. Second, selection bias is always possible in this type of survey, but in our opinion, it was reduced to a minimum by selecting physicians at random, including patients consecutively, and obtaining a high participation rate. In the context of our study, where patients were present in the waiting room, the selection biases usually described for electronic surveys in general practice do not apply [32]. Third, the study was carried out in only 1 region of France and only with primary care patients. The results obtained could have been different for other regions or countries, or for other study populations (physicians or patients visiting specialists). Finally, the use of simple randomization resulted in an unequal allocation of clusters across the study groups. While this does not affect the validity of randomization, it may have slightly reduced the power to detect differences between groups.

### Conclusions

In conclusion, by recruiting patients in the waiting room with the help of a research assistant providing support to participants, we obtained a participation rate of over 80% in all groups. Neither the choice of response mode (ie, single or mixed, with or without a QR code) nor the use of incentives markedly influenced participation or completion rates. Future research would be useful to compare these rates with and without the presence of a research assistant in the waiting room to answer questions from participants. In terms of feasibility, the use of mixed options, including innovative methods such as QR codes, to offer participants the opportunity to choose, might be a relevant strategy, despite the results of our study showing no superiority in terms of participation and completion rates.

## Data Availability

The data associated with this article are available from the corresponding author upon reasonable request.

## Appendix

Multimedia Appendix 1 Questionnaire used in the study.
