# Supplementary material for: Increasing Participation and Completion Rates in Questionnaire Surveys of Primary Care Patients: Cluster-Randomized Study
Source: Interact J Med Res. 2025 Feb 25;14:e67981. doi: 10.2196/67981 (PMC11897665; doi:10.2196/67981)
Supplement: Multimedia Appendix 1 [file ijmr_v14i1e67981_app1.docx]

**Questionnaire**

**Merci de prendre connaissance de la lettre d’information et de signer le consentement avant de répondre à ces questionnaires.**

**
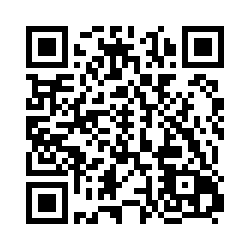
**

**Partie 1 : A propos de vous 1**

**Partie 2 : A propos de votre attachement à la viande 2**

**Partie 3 : Questions diverses 3**

**Partie 3 (suite) : Questions diverses 4**

**Partie 4 : A propos de vos intentions 5**

**Partie 1 : A propos de vous**

**1/ Quelle est votre année de naissance ? (YYYY)**

**2/ Etes-vous (cocher la réponse correcte)**

☐ Un homme ☐ Une femme ☐ Non binaire

**3/ Quel est votre code postal de résidence ?**  _______

**4/ Quelle est votre catégorie socio professionnelle (selon INSEE) ? (cochez la réponse correcte)**

☐ Agriculteurs/Agricultrices exploitant.es

☐ Artisan.es commerçant.es; chef.fes d’entreprise de plus de 10 personnes

☐ Cadres et professions intellectuelles supérieures*

☐ Professions intermédiaires**

☐ Employé.es***

☐ Ouvriers/Ouvrières****

☐ Retraité.e

☐ Sans activité

*/**/***/**** : se référer à la fiche INSEE disponible auprès de l’investigateur.

| **Partie 2 : A propos de votre attachement à la viande**  Merci de cocher la proposition qui vous correspond le mieux pour chacune des questions. | **Pas du tout d’accord** | **Plutôt pas d’accord** | **Ni d’accord, ni pas d’accord** | **Plutôt d’accord** | **Tout à fait d’accord** |
| --- | --- | --- | --- | --- | --- |
| **1/ Manger de la viande est un des bons plaisirs de la vie** | ☐ | ☐ | ☐ | ☐ | ☐ |
| **2/ Rien ne peut remplacer la viande dans mon alimentation** | ☐ | ☐ | ☐ | ☐ | ☐ |
| **3/ Du fait de notre place dans la chaîne alimentaire, nous avons le droit de manger de la viande** | ☐ | ☐ | ☐ | ☐ | ☐ |
| **4/ Je me sens mal à l’idée de manger de la viande** | ☐ | ☐ | ☐ | ☐ | ☐ |
| **5/ J’adore les repas avec de la viande** | ☐ | ☐ | ☐ | ☐ | ☐ |
| **6/ Manger de la viande est irrespectueux de la vie et de l’environnement** | ☐ | ☐ | ☐ | ☐ | ☐ |
| **7/ Manger de la viande est un droit incontestable de chaque personne** | ☐ | ☐ | ☐ | ☐ | ☐ |
| **8/ Rien ne vaut un bon steak** | ☐ | ☐ | ☐ | ☐ | ☐ |
| **9/ Une alimentation sans viande me conviendrait très bien** | ☐ | ☐ | ☐ | ☐ | ☐ |
| **10/ Je raffole de la viande** | ☐ | ☐ | ☐ | ☐ | ☐ |
| **11/ Si je ne pouvais pas manger de viande, je me sentirais faible** | ☐ | ☐ | ☐ | ☐ | ☐ |
| **12/ Si on m’obligeait à cesser de manger de la viande, je serais triste** | ☐ | ☐ | ☐ | ☐ | ☐ |
| **13/ La viande me fait penser à des maladies** | ☐ | ☐ | ☐ | ☐ | ☐ |
| **14/ En mangeant de la viande, je pense à la mort et à la souffrance des animaux** | ☐ | ☐ | ☐ | ☐ | ☐ |
| **15/ Manger de la viande est une pratique naturelle** | ☐ | ☐ | ☐ | ☐ | ☐ |
| **16/ Manger de la viande est une pratique indiscutable** | ☐ | ☐ | ☐ | ☐ | ☐ |
| **17/ Je ne me vois pas ne pas manger de viande régulièrement** | ☐ | ☐ | ☐ | ☐ | ☐ |

**Partie 3 : Questions diverses**

Pour chaque question, merci de cocher le numéro correspondant le mieux à ce que vous ressentez (sur une échelle de 1 à 7).

| **1)**  **Vous avez le sentiment que vous ne vous souciez pas réellement de ce qui se passe autour de vous** | | | | | | | | |
| --- | --- | --- | --- | --- | --- | --- | --- | --- |
| **Très rarement ou jamais** | 1  ☐ | 2  ☐ | 3  ☐ | 4  ☐ | 5  ☐ | 6  ☐ | 7  ☐ | **Très souvent** |
| **2)**  **Vous est-il arrivé dans le passé d’être surpris.e par le comportement de gens que vous pensiez connaître très bien ?** | | | | | | | | |
| **Jamais** | 1  ☐ | 2  ☐ | 3  ☐ | 4  ☐ | 5  ☐ | 6  ☐ | 7  ☐ | **Toujours** |
| **3)** **Est-il arrivé que des gens sur lesquels vous comptiez vous déçoivent ?** | | | | | | | | |
| **Jamais** | 1  ☐ | 2  ☐ | 3  ☐ | 4  ☐ | 5  ☐ | 6  ☐ | 7  ☐ | **Toujours** |
| **4)** **Jusqu’à maintenant, votre vie n’a eu :** | | | | | | | | |
| **Aucun but ni objectif** | 1  ☐ | 2  ☐ | 3  ☐ | 4  ☐ | 5  ☐ | 6  ☐ | 7  ☐ | **Des buts et des objectifs très clairs** |
| **5)** **Avez-vous le sentiment que vous êtes traité.e injustement ?** | | | | | | | | |
| **Très souvent** | 1  ☐ | 2  ☐ | 3  ☐ | 4  ☐ | 5  ☐ | 6  ☐ | 7  ☐ | **Très rarement ou jamais** |
| **6)** **Avez-vous le sentiment que vous êtes dans une situation inconnue et que vous ne savez pas quoi faire ?** | | | | | | | | |
| **Très souvent** | 1  ☐ | 2  ☐ | 3  ☐ | 4  ☐ | 5  ☐ | 6  ☐ | 7  ☐ | **Très rarement ou jamais** |
| **7)** **Faire les choses que vous faites quotidiennement est une source :** | | | | | | | | |
| **De plaisir et de satisfaction profonde** | 1  ☐ | 2  ☐ | 3  ☐ | 4  ☐ | 5  ☐ | 6  ☐ | 7  ☐ | **De souffrance et d’ennui** |
| **8)** **Avez-vous des idées ou des sentiments confus ?** | | | | | | | | |
| **Très souvent** | 1  ☐ | 2  ☐ | 3  ☐ | 4  ☐ | 5  ☐ | 6  ☐ | 7  ☐ | **Très rarement ou jamais** |

| **9) Vous arrive-t-il d’avoir des sentiments intimes que vous préféreriez ne pas avoir ?** | | | | | | | | |
| --- | --- | --- | --- | --- | --- | --- | --- | --- |
| **Très souvent** | 1  ☐ | 2 ☐ | 3  ☐ | 4  ☐ | 5  ☐ | 6  ☐ | 7  ☐ | **Très rarement ou jamais** |
| **10) Beaucoup de gens (même s’ils ont beaucoup de caractère) se sentent parfois de pauvres cloches. Avez-vous déjà eu ce sentiment dans le passé ?** | | | | | | | | |
| **Très souvent** | 1  ☐ | 2  ☐ | 3  ☐ | 4  ☐ | 5  ☐ | 6  ☐ | 7  ☐ | **Très rarement ou jamais** |
| **11) Quand quelque chose arrive, vous trouvez généralement que :** | | | | | | | | |
| **Vous surestimez ou sous-estimez son importance** | 1  ☐ | 2  ☐ | 3  ☐ | 4  ☐ | 5  ☐ | 6  ☐ | 7  ☐ | **Vous voyez les choses dans de justes proportions** |
| **12) Avez-vous le sentiment que les choses que vous faites dans la vie quotidienne ont peu de sens ?** | | | | | | | | |
| **Très souvent** | 1  ☐ | 2  ☐ | 3  ☐ | 4  ☐ | 5  ☐ | 6  ☐ | 7  ☐ | **Très rarement ou jamais** |
| **13) Vous avez le sentiment que vous n’êtes pas sûr.e de vous maîtriser** | | | | | | | | |
| **Très souvent** | 1  ☐ | 2  ☐ | 3  ☐ | 4  ☐ | 5  ☐ | 6  ☐ | 7  ☐ | **Très rarement ou jamais** |
| **14) Quand vous pensez à votre vie, très souvent :** | | | | | | | | |
| **Vous ressentez combien**  **il est bon d’être en vie** | 1  ☐ | 2  ☐ | 3  ☐ | 4  ☐ | 5  ☐ | 6  ☐ | 7  ☐ | **Vous vous demandez pourquoi, au fond, vous existez** |

| **Partie 3 : Questions diverses (suite)** | **Oui souvent** | **Oui parfois** | **Non** |
| --- | --- | --- | --- |
| **15/ Voyez-vous une solution aux problèmes et aux difficultés que d’autres trouvent sans espoir ?** | ☐ | ☐ | ☐ |
| **16/ Percevez-vous votre vie comme une source de satisfaction personnelle ?** | ☐ | ☐ | ☐ |
| **17/ Ressentez-vous que les choses qui vous arrivent sont difficiles à comprendre ?** | ☐ | ☐ | ☐ |

| **Partie 4 : A propos de vos intentions**  Merci de cocher la proposition qui vous correspond le mieux pour chacune des questions. | **Pas du tout d’accord** | **Pas d’accord** | **Plutôt pas d’accord** | **Indifférent** | **Plutôt d’accord** | **D’accord** | **Tout à fait d’accord** |
| --- | --- | --- | --- | --- | --- | --- | --- |
| **1/** Je **ne** projette **pas** de manger **moins** de viande dans les **6** **prochains** **mois** | ☐ | ☐ | ☐ | ☐ | ☐ | ☐ | ☐ |
| **2/** Je projette de manger **moins** de viande **d’ici** **6 mois** | ☐ | ☐ | ☐ | ☐ | ☐ | ☐ | ☐ |
| **3/** Je projette de manger **moins** de viande **d’ici** **1 mois** | ☐ | ☐ | ☐ | ☐ | ☐ | ☐ | ☐ |
| **4/** J’ai **déjà commencé** à manger **moins** de viande dans les **6 derniers** **mois** | ☐ | ☐ | ☐ | ☐ | ☐ | ☐ | ☐ |
| **5/** J’ai **commencé** à manger **moins** de viande depuis **plus** **de 6 mois** | ☐ | ☐ | ☐ | ☐ | ☐ | ☐ | ☐ |
| **_ _ _ _ _ _ _ _ _ _ _ _ _ _ _ _ _ _ _ _** | **_** | **_** | **_** | **_** | **_** | **_** | **_** |
| **6/** Je **ne** projette **pas** de manger **plus (+)** de viande dans les **6 prochains** **mois** | ☐ | ☐ | ☐ | ☐ | ☐ | ☐ | ☐ |
| **7/** Je projette de manger **plus** de viande **d’ici 6 mois** | ☐ | ☐ | ☐ | ☐ | ☐ | ☐ | ☐ |
| **8/** Je projette de manger **plus** de viande **d’ici 1 mois** | ☐ | ☐ | ☐ | ☐ | ☐ | ☐ | ☐ |
| **9/** J’ai **déjà commencé** à manger **plus** de viande dans les **6 derniers mois** | ☐ | ☐ | ☐ | ☐ | ☐ | ☐ | ☐ |
| **10 /** J’ai **commencé** à manger **plus** de viande depuis **plus de 6 mois** | ☐ | ☐ | ☐ | ☐ | ☐ | ☐ | ☐ |
|  |  |  |  |  |  |  |  |
